# 4D Printing of Multifunctional and Biodegradable PLA‐PBAT‐Fe_3_O_4_ Nanocomposites with Supreme Mechanical and Shape Memory Properties

**DOI:** 10.1002/marc.202400661

**Published:** 2024-10-14

**Authors:** Mohammad Amin Yousefi, Davood Rahmatabadi, Majid Baniassadi, Mahdi Bodaghi, Mostafa Baghani

**Affiliations:** ^1^ School of Mechanical Engineering College of Engineering University of Tehran Tehran 1417614411 Iran; ^2^ Department of Engineering School of Science and Technology Nottingham Trent University Nottingham NG11 8NS UK

**Keywords:** 4D Printing, Fe_3_O_4_ nanoparticles, magneto‐responsive structures, material extrusion, shape memory polymers

## Abstract

4D printing magneto‐responsive shape memory polymers (SMPs) using biodegradable nanocomposites can overcome their low toughness and thermal resistance, and produce smart materials that can be controlled remotely without contact. This study presented the development of 3D/4D printable nanocomposites based on poly (lactic acid) (PLA)‐poly (butylene adipate‐co‐terephthalate) (PBAT) blends and magnetite (Fe_3_O_4_) nanoparticles. The nanocomposites are prepared by melt mixing PLA‐PBAT blends with different Fe_3_O_4_ contents (10, 15, and 20 wt%) and extruded into granules for material extrusion 3D printing. The morphology, dynamic mechanical thermal analysis (DMTA), mechanical properties, and shape memory behavior of the nanocomposites are investigated. The results indicated that the Fe_3_O_4_ nanoparticles are preferentially distributed in the PBAT phases, enhancing the storage modulus, thermal stability, strength, elongation, toughness, shape fixity, and recovery of the nanocomposites. The optimal Fe_3_O_4_ loading is found to be 10 wt%, as higher loadings led to nanoparticle agglomeration and reduced performance. The nanocomposites also exhibited fast shape memory response under thermal and magnetic activation due to the presence of Fe_3_O_4_ nanoparticles. The 3D/4D printable nanocomposites demonstrated multifunctional multi‐trigger shape‐memory capabilities and potential applications in contactless and safe actuation.

## Graphical abstract

This study pioneers 4D printing by developing multifunctional, biodegradable PLA‐PBAT‐Fe3O4 nanocomposites with superior mechanical and shape memory properties. While 10% Fe3O4 offers optimal mechanical performance, higher concentrations accelerate bi‐stimuli (thermal and magnetic) shape recovery, making these materials ideal for advanced, remote‐controlled biomedical applications.

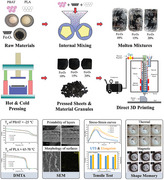

## 1 Introduction

Fused deposition modeling (FDM), a type of 3D printing technology, has become more widely available to homes and small businesses.^[^ ^1^, ^2^ ^]^ As FDM printing has shifted from prototyping to small‐scale production, 3D printable materials must be adapted to meet the increasing requirements for printed products' mechanical and thermal properties.^[^ ^3^, ^4^ ^]^

Poly (lactic acid) (PLA) is known as the most used thermoplastic in FDM 3D printing. Also, in today's world, PLA is the most commonly preferred biodegradable polymer, a renewable, bio‐based, biodegradable, and aliphatic linear polyester derived from sugar. It has desirable properties such as high mechanical strength, transparency, and non‐toxicity.^[^ ^5^, ^6^, ^7^ ^]^ However, PLA also has some shortcomings, such as low melt flow index, low thermal stability, low service temperatures/heat deflection points, and brittleness.^[^ ^8^, ^9^, ^10^ ^]^ In order to overcome these problems, scientists have blended PLA with ductile polymers, such as elastomers and PBAT, to enhance its toughness and processability. These blends also reduce the need for plasticizers, which can impair the composability of PLA.^[^ ^11^, ^12^, ^13^ ^]^ One of the most suitable polymers to blend with PLA is PBAT, a petroleum‐based aliphatic‐aromatic copolymer that exhibits high ductility, robustness, and melt strength.^[^ ^14^, ^15^, ^16^, ^17^ ^]^ The PLA‐PBAT blend is a feasible option in this respect, as it has outstanding physicochemical and mechanical characteristics as well as biodegradability.^[^ ^18^, ^19^, ^20^, ^21^ ^]^

The following sections review some recent research on 3D and 4D printing of PLA‐PBAT. Andrzejewski et al.^[^ ^14^ ^]^ reinforced PLA with PBAT (10%, 20%, 30%) for FDM 3D printing. With 30% PBAT (the optimal percentage), impact resistance increased from 30 J m^−1^ to over 700 J m^−1^ without compromising thermal properties.

Mathew et al.^[^ ^22^ ^]^ proposed an eco‐friendly alternative to conventional fossil fuel‐derived filaments from a PLA matrix toughened with varying concentrations of PBAT. The addition of 30% PBAT notably improved toughness and impact resistance while maintaining thermal stability. SEM analysis confirmed changes in fracture morphology, explaining the enhanced toughness.

Sritham et al.^[^ ^23^ ^]^ reported that PLA/PBAT blends exhibited improved elastic modulus with a 40% PLA concentration. However, the ultimate tensile strength remained consistent across different PLA concentrations. Elongation at break (E_b_) reached a minimum of 13% when PLA content exceeded 30%.

Research by Cardoso et al.^[^ ^24^ ^]^ assessed the impact of print settings on PLA‐PBAT blends using a Design of Experiment approach. Finer settings improved flexural strength by reducing porosity, while PBAT increased tear resistance despite reduced stiffness. Thermogravimetric analysis (TGA) confirmed thermal stability, and SEM revealed that lower settings strengthened filament bonds and minimized voids, enhancing mechanical robustness.

Chen et al.^[^ ^25^ ^]^ examined degradation of PLA‐PBAT prepared via solution casting. Results showed that while Young's modulus remained stable, tensile strain significantly decreased with degradation, indicating reduced mechanical performance.

By combining PLA and PBAT with 3D printing technology, smart materials with shape memory effect (SME) may be created, which might result in the creation of 4D structures. These smart materials offer self‐assembly, multiple functions, self‐repair capabilities, and exhibit time dependence and predictability.^[^ ^26^, ^27^, ^28^ ^]^ SMPs have the ability to return from predetermined temporary shapes to their original forms when an external stimulus is applied, including water, thermal, magnetic, electric, optical, and other activation methods.^[^ ^29^, ^30^ ^]^ Iron (III) oxide (Fe_3_O_4_) particles are among the commonly employed fillers for achieving magnetic‐active shape memory effects.^[^ ^31^ ^]^ The ability to induce SME through thermomagnetic and electromagnetic methods allows embedded SMP devices to be activated remotely via an external magnetic field, thus enabling non‐contact control.^[^ ^32^ ^]^ Such capabilities open up application opportunities, including tissue engineering scaffolds,^[^ ^33^, ^34^ ^]^ tracheal stents,^[^ ^35^, ^36^ ^]^ drug delivery systems,^[^ ^37^ ^]^ and implant devices.^[^ ^38^, ^39^, ^40^ ^]^

Zhang et al.^[^ ^41^ ^]^ explored the impact of Fe_3_O_4_ on the shape memory behavior and thermal properties of PLA/Fe_3_O_4_ composites. The composite's shape memory was analyzed with various stimuli. Quick shape recovery within 5 s occurred in the hot water bath, while magnetic stimulation took 8, 10, and 14 s for Fe_3_O_4_ concentrations of 10%, 15%, and 20%, respectively. The glass transition temperature (T_g_) values increased with higher Fe_3_O_4_ concentrations, which are ≈65 °C, indicating improved thermal stability. It was found that 20% of Fe_3_O_4_ exhibited the highest and most efficient shape memory properties and thermal stability compared to lower concentrations.

Liu et al.^[^ ^42^ ^]^ developed magneto‐responsive SMPs based on PLA, thermoplastic polyurethane (TPU), and Fe_3_O_4_ particles for 4D printing applications. The Fe_3_O_4_ addition enhanced PLA crystallinity, achieving shape fixity and recovery ratios of ≈100% and >91%, respectively, with rapid magnetic response times (as short as 40 s) and robust tensile strength (up to 54 MPa).

In this study, we aimed to elevate the development and fabrication of magneto‐responsive SMP nanocomposites derived from blends of PLA‐PBAT reinforced with varying concentrations of magnetite nanoparticles (Fe_3_O_4_). Unlike previous research on various PLA‐based blends, our approach introduces the unique combination of PLA and PBAT, enhancing flexibility, microstructure, and mechanical properties while maintaining biodegradability, an important advantage over non‐biodegradable TPU. PBAT's compatibility with PLA ensures that the environmental benefits of PLA are preserved. The blend is melt‐mixed into granules for FDM 3D printing, a key innovation that avoids the additional thermomechanical cycle of filament‐based methods, preserving material structure with greater accuracy. Analyses were conducted to investigate the effects of Fe_3_O_4_ nanoparticle additions on dynamic mechanical properties, tensile mechanical performance, printability, morphology, and actuation induced by both thermal and magnetic stimuli. Collectively, the resulting nanocomposites exhibit superior load‐bearing capacity and rapid magnetic actuation compared to traditional PLA‐based blends. By addressing a critical gap in the literature, this study provides new insights into the magneto‐responsiveness and fabrication of PLA‐PBAT‐Fe_3_O_4_ nanocomposites via a pneumatic direct 3D printing process, providing a significant contribution to the advancement of multifunctional, next‐generation materials, primarily used in biomedical applications.

## 2 Materials and Methods

### 2.1 Materials

The raw materials used in this study to prepare the PLA‐PBAT blends included 2003D‐grade PLA granules from Nature Works, with a molecular weight of 42,700 g mol^−1^, and KD 1024‐grade PBAT granules, with a molecular weight of 52,100 g mol^−1^, sourced from Zhuhai Wango Chemical Co. Prior to processing, the raw materials were dehumidified in an oven at 60 °C for PLA and 40 °C for PBAT for a duration of 9 h. Magnetic iron oxide particles (Fe_3_O_4_) with an average particle size of 20–30 nm were obtained from US Nano.

### 2.2 Nanocomposites Preparation

**Figure 1:**
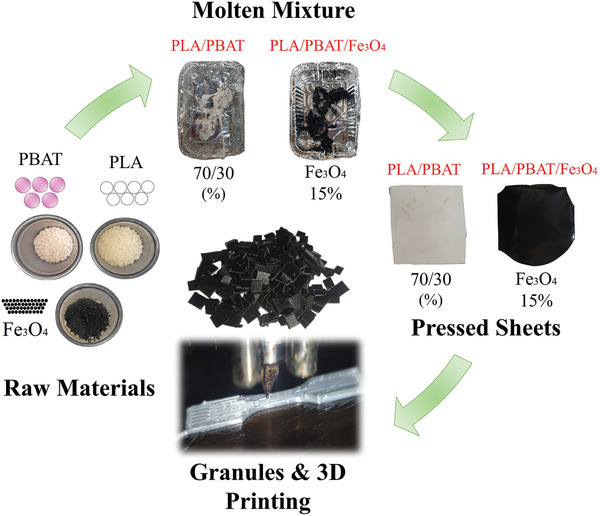
Process of nanocomposite preparation.

In the preparation of nanocomposites, the commingling of polymers PLA and PBAT was performed using a melt mixing procedure, adhering to constant PLA‐PBAT weight ratios of (70/30). Additionally, according to **Table** **1**, PLA, PBAT, and Fe_3_O_4_ were combined in different weight percentages, resulting in PLA‐PBAT‐Fe_3_O_4_ ratios of (70/30/10phr, 70/30/15phr, and 70/30/20phr). The raw materials were melted and homogenized using a Brabender internal mixer with a 60 cc capacity, operating at a temperature of 190 °C and a rotational speed of 100 rpm. After a 2‐min interval from the onset of PLA's melting phase, PBAT raw materials were introduced to the fully molten PLA. The blend was processed for an additional 10 min to ensure their complete assimilation within the polymer matrix. The materials produced were lumpy blends. Following the mixing phase, a two‐step pressing process was employed. First, the material was heated to 200 °C for 6 min using a hot press, and subsequently cooled under 60 kPa pressure in a cold press for 4 min. After this, the formed sheets were granulated to prepare them for 3D printing. This process is depicted in **Figure** **1**.

**Table 1:** Compositions of PLA‐PBAT and PLA‐PBAT‐Fe_3_O_4_.

Sample	PLA	PBAT	Fe_3_O_4_
	g	g	g
PLA‐PBAT	42	18	0
PLA‐PBAT‐F10	42	18	6
PLA‐PBAT‐F15	42	18	9
PLA‐PBAT‐F20	42	18	12

### 2.3 3D Printing

**Figure 2:**
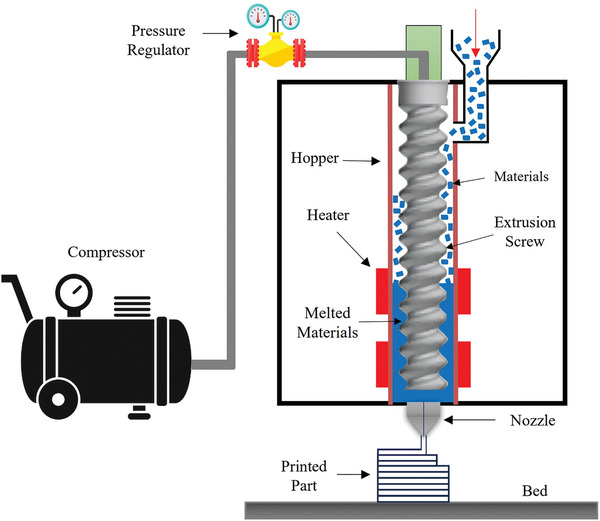
The schematic of the direct FDM 3D printer utilized for this experiment.

**Figure 3:**
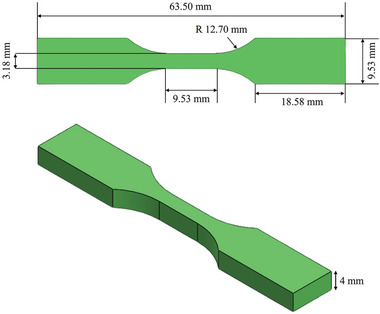
Printed tensile samples: ASTM D638‐V standard dimensions (unit: mm).

The PLA‐PBAT‐Fe_3_O_4_ specimens were 3D printed using a new‐generation FDM 3D printer. The schematic illustration of the printer used in this experiment is shown in **Figure** **2**. Unlike conventional printers that rely on filament extraction, this system directly feeds material granules into the thermal chamber via a pneumatic system. Direct granule printing bypasses the filamentation step, reducing the number of thermomechanical processes the material undergoes. This reduction could enhance the material's mechanical, thermal, and shape memory properties due to the significant effects of thermal and stress history on polymer characteristics. In this method, the granules are melted within the cartridge and achieve the appropriate rheological properties in a semi‐molten state before being directed into the nozzle through air pressure for printing. To ensure a stable output flow and prevent nozzle clogging due to the addition of Fe_3_O_4_, two key parameters, nozzle temperature, and air pressure, were adjusted, as the printing parameters vary with different Fe_3_O_4_ concentrations. The temperature was varied within a range of 200 ± 10 °C, while the pressure behind the nozzle was controlled between 3–8 bar. This allowed for effective rheological control and consistent material flow across all composite variations. The FDM process involves various parameters, as listed in **Table** **2**, that greatly influence both production efficiency and the characteristics of the manufactured part. The printing temperature for the PLA‐PBAT blend was initially determined based on the PLA's printing temperature. However, it was lowered to accommodate the reduced melting point caused by the addition of PBAT, ensuring optimal printing conditions. Additionally, The dogbone‐shaped samples, measuring and intended for the tensile test, were fabricated using 3D printing technology, as shown in **Figure** **3**.^[^ ^43^ ^]^

**Table 2:** 3D printing conditions of printed samples.

Parameters	Conditions
Nozzle temperature ([C]	200 ± 10
Bed temperature [°C]	60
Printing speed [mm ^−1^s]	300
Raster	0/90
Number of walls	2
Nozzle diameter [mm]	0.6
Layer thickness [mm]	0.4

### 2.4 DMTA

An analysis that combines thermal and mechanical properties was performed to study the materials’ fundamental elastic characteristics, including their storage modulus and the relationship between loss and storage moduli. Rectangular samples with dimensions of 40 mm × 10 mm × 1.5 mm were prepared and tested according to the standard DMTA procedure. This was done using a Mettler Toledo dynamic thermomechanical device from Switzerland. During the tests, a steady temperature increase of 5 °C min^−1^ heating rate was maintained within a temperature range of −100 to 100 °C. The level of deformation applied to the samples was kept constant, and the stress was applied at a steady frequency of 1 Hz.

### 2.5 Mechanical Properties

Investigations were conducted to determine how the print settings affect the bending qualities, such as Young's modulus, Ultimate Tensile Strength (UTS), and Elongation, for parts printed utilizing FDM. The tensile strength of the 3D‐printed test pieces was measured using a standard material testing device, Santam, which is designed with a tensile testing apparatus and a load cell capable of 500 kN. This machine was operated at a uniform rate of 3 mm min^−1^ for testing all samples, in alignment with the specifications of ASTM D638‐V, and a gage length setting of 9.53 mm was used. Each test was repeated at least three times to verify its repeatability.

### 2.6 Morphology and Printability

The morphological and printability analysis of the tensile fractured surface of the composite samples was conducted by employing SEM (Tescan Vega Machine). Prior to examination, the cross‐sectional surface was acquired through a brittle fracture in a liquid nitrogen environment, in accordance with standard laboratory procedures, and the fractured filament samples were coated with gold and subsequently dried at room temperature.

### 2.7 4D Printing

**Figure 4:**
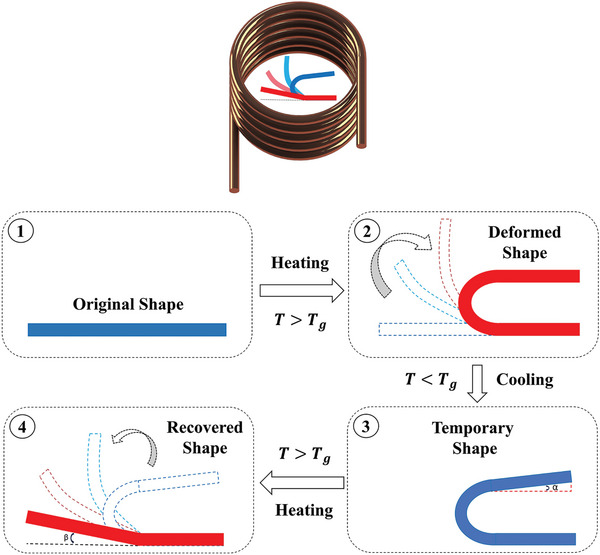
Shape recovery analysis process.

The PLA‐PBAT‐Fe_3_O_4_ nanocomposites were evaluated for the magnetic‐induced shape recovery effect using a high‐frequency induction with a magnetic induction coil. Rectangular specimens measuring [40 × 10 × 1.5 mm^3^] were employed for the tests. The magnetic induced and the shape memory process are depicted in **Figure** **4**. First, the rectangularly shaped sample underwent a heating process and was loaded to 80 °C (above T_g_ of nanocomposites) in a hot water bath. Then, it was cooled in a water bath to obtain a fixed shape. The external force was continuously applied until the temperature of the sample was reduced below its T_g_, resulting in hardening. The angle achieved was recorded as α. The specimen, now in a fixed shape, was placed in a magnetic field and into the water bath to induce reheating. Once the specimen temperature exceeded its T_g_, the sample was unfolded and regained its initial shape. The unrecovered angle of the sample after the experimental process was marked as β. A video camera recorded the shape recovery process of the specimens. The formulas for calculating the shape fixed ratio (R_f_) and shape recovery ratio (R_r_) are given by equations (1) and (2).

(1) $$R_{f}\left(\%\right)=\frac{\theta_{fixed}}{180^{\circ }}\times 100$$

(2) $$R_{r}\left(\%\right)=\frac{\theta_{recoverd}}{180^{\circ }}\times 100$$

The angle θ_*recoverd*_ represents the deformation angle when the original shape is restored, which is $180^{\circ }-\beta$, while the angle θ_*fixed*_ is equal to $180^{\circ }-\alpha$, denoting the supplementary angle of α at which the specimen of fixed shape returns after the external stimuli are removed, and cooling is applied.

## 3 Results and Discussion

### 3.1 DMTA

**Figure 5:**
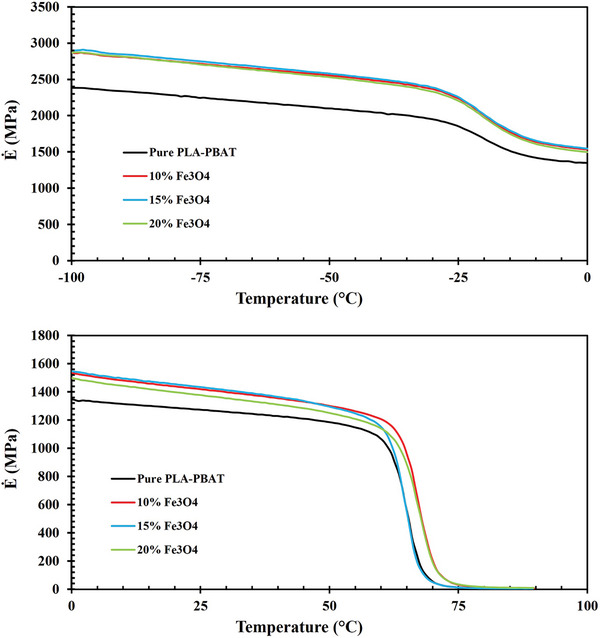
Temperature‐dependent storage modulus changes with varied Fe_3_O_4_ contents from −100 to 100 °C.

**Figure 6:**
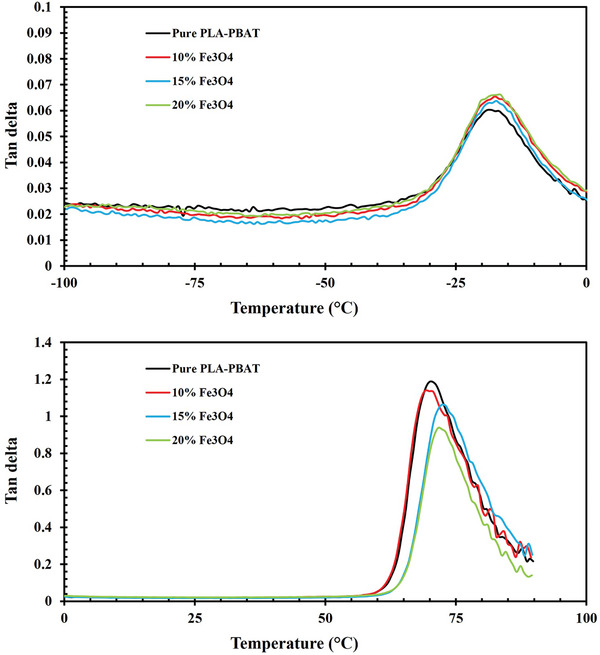
Temperature‐dependent Tan δ changes with varied Fe_3_O_4_ contents from −100 to 100 °C.

As an immiscible polymer blend, the PLA‐PBAT composition displays discrete thermal transitions corresponding to the individual components in the storage modulus and Tan δ diagrams (**Figures** **5** and **6**). PLA's T_g_ is higher than that of PBAT, attributed to the more ordered and rigid structure of PLA chains compared to the flexible nature of PBAT chains. Below the T_g_, the material is in a glassy state, characterized by a high storage modulus due to restricted chain mobility.^[^ ^44^ ^]^ In this region, all Fe_3_O_4_‐reinforced blends exhibit a storage modulus between 2500 and 3000 MPa, surpassing that of the neat PLA/PBAT blend. As the T_g_ is reached, the storage modulus decreases, reflecting an increase in chain mobility and material flexibility. This reduction occurs twice in the blends, indicating two distinct T_g_ points, corresponding to two different phases.

The sharp decrease at ≈−25 °C correlates with the T_g_ of the PBAT phase. The more intense decline in modulus initiating at roughly 60 °C aligns with the T_g_ of PLA. The PBAT material displays a glassy state within the temperature range of −100 °C to ≈−25 °C, with Fe_3_O_4_‐15% showing the highest and most consistent storage modulus value of 2910 MPa. PLA possesses a glass transition temperature in the range of 50 to 70 °C (depending on PLA grade) and a melting point temperature between 180 and 220 °C.^[^ ^45^, ^46^, ^47^ ^]^ In this case, the T_g_ of the PLA phase becomes apparent at 65–70 °C, which contributes to the strength and extent of the blend transition peak due to its higher relative volume fraction compared to PBAT.

The addition of magnetite nanoparticles induces noticeable enhancements in storage modulus across the entire temperature sweep. Specifically, at room temperature in the glassy state, modulus values show incremental improvements of ≈12%, 13%, and 8% for the 10%, 15% and 20% Fe_3_O_4_ formulations, respectively, compared to the pure PLA‐PBAT. The impact of varying percentages of magnetite nanoparticles on storage modulus persists up to ≈75 °C, marking the transition region's end and the rubbery state's onset. Notably, the Fe_3_O_4_‐15% formulation deviates from this trend, aligning with the pure PLA‐PBAT behavior at ≈65 °C and exhibiting distinct characteristics compared to other Fe_3_O_4_ percentages. The glass‐rubber relaxation region is responsible for the significant decrease in storage modulus from ≈1048 MPa, 1213 MPa, 1156 MPa, and 1132 MPa to 46 MPa, 256 MPa, 50 MPa, and 162 MPa MPa, respectively, for Pure PLA‐PBAT, 10%, 15%, 20% of Fe_3_O_4_ between 60–70 °C. Relatively more minor enhancements are observed in the rubbery state, suggesting that the strengthening effect primarily originated from the constraints imposed by the nanoparticles on the movement of amorphous chain segments.

It should be noted that immiscible polymers with proximate T_g_ values can exhibit improved interfacial adhesion and compatibility through molecular interdiffusion across the boundary.^[^ ^48^ ^]^ Here, the sizable T_g_ discrepancy of ≈90 °C between PLA (65–70 °C) and PBAT (−25 °C) signifies their relatively poor miscibility. In the glassy state below each constituent T_g_, polymer chain segment mobility is heavily restricted, conferring maximal stiffness as evidenced by the highest storage modulus values in the diagrams. The glass‐to‐rubber relaxation process spans each transition region until a plateau emerges, marking the onset of rubbery state viscous flow behavior.^[^ ^49^ ^]^

Tan δ is used to quantify the ability of a viscoelastic material to dissipate energy, showing the relationship between its viscous and elastic properties.^[^ ^50^ ^]^ Tan δ represents the ratio of the loss modulus, which indicates the material's viscous properties, to the storage modulus, which reflects its elastic properties.^[^ ^49^ ^]^ This parameter is essential for assessing a material's capacity to absorb and release energy when subjected to external forces, a key factor in analyzing shape memory behavior.

As the Fe_3_O_4_ content increases, the Tan δ peaks show a slight downward shift, indicating that the inorganic particulate phase impedes the molecular relaxations in both polymer constituents by introducing additional interfaces and confined micro‐domains that the polymer chains must navigate. Peaks in Tan δ are associated with polymer chain movements and phase transitions, and the observed shift in peak values, along with higher peak temperatures, suggests a reduction in chain mobility. This reduction is likely caused by the Fe_3_O_4_ nanoparticles, which act as obstacles, restricting molecular motion and increasing the temperature required for the polymer chains to transition between phases.

Consequently, the embedded magnetite nanoparticles impart stepwise improvements to the stiffness, thermomechanical stability, and restriction of molecular motions in the PLA/PBAT matrix. Such observations align with established nanocomposite theory, where the high interfacial area facilitates strong polymer‐filler interactions. An inorganic phase loading threshold appears to occur between 15% and 20%, beyond which modulus enhancements become significant. Notably, the transitions in the nanocomposites, particularly the glass‐to‐rubber transition, play a crucial role in shape memory behavior, as the temperature at which they occur acts as the switching point for shape recovery. Conversely, drawbacks such as embrittlement and challenging processability would be expected at the highest loadings examined.

### 3.2 Printability

**Figure 7:**
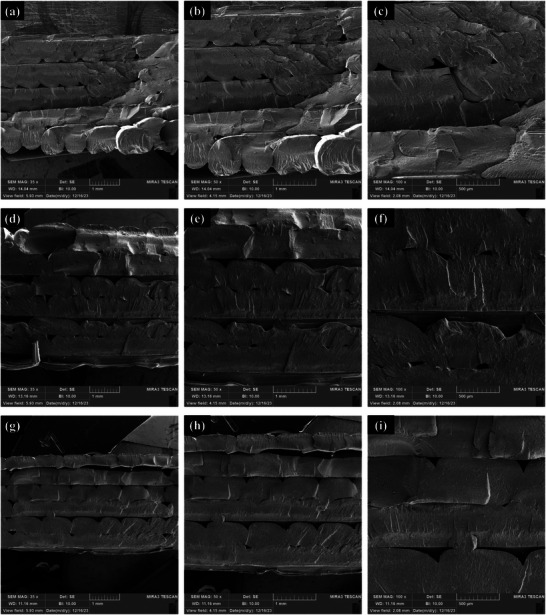
SEM analysis of PLA‐PBAT nanocomposites with different weight percentages of nanoparticles: a–c) 10%, d–f) 15%, and g–i) 20% at various magnifications (35x, 50x, 100x).

The cohesion between layers plays a critical role in FDM 3D printing, as enhancing interlayer adhesion has the potential to positively impact the mechanical characteristics of printed objects.^[^ ^51^, ^52^ ^]^ Furthermore, printing parameters significantly influence the quality of interlayer bonding and the formation of microvoids. The PLA‐PBAT‐Fe_3_O_4_ blends were printed under consistent, optimized printing conditions to minimize these effects. The printability characteristics of fractured surfaces from 3D‐printed PLA‐PBAT‐Fe_3_O_4_ nanocomposites are meticulously examined utilizing SEM in **Figure** **7**. The nanocomposites, comprising varying concentrations of Fe_3_O_4_ (10%, 15%, and 20% by weight), are scrutinized to elucidate the influence of Fe_3_O_4_ on the printability and printing quality of the PLA‐PBAT matrix. SEM micrographs captured at magnifications of 35x, 50x, and 100x revealed discernible differences in surface topography correlated with the Fe_3_O_4_ content.

The first set of images (a, b, and c) corresponds to the PLA‐PBAT‐Fe_3_O_4_‐10% blend. This composition has the lowest Fe_3_O_4_ content and the highest PLA‐PBAT content among the three blends. The images reveal that the surface morphology of this blend is relatively smooth and uniform, with consistent striations along the printing direction. This indicates that the printability of this composite is high, as the PLA‐PBAT provides rigidity and stability to the printed structure. This blend's layer adhesion and structural integrity are also good, as there are no visible gaps or cracks on the surface. The low Fe_3_O_4_ content does not significantly affect the printing performance of PLA‐PBAT, but it may impart some magnetic properties to the composite.

Figure 7 (d, e, and f) reveals a different nanocomposite of PLA‐PBAT‐Fe_3_O_4_‐15%, with more Fe_3_O_4_ and constant PLA‐PBAT than the previous one. Unlike the smooth and uniform one of the first composites, this nanocomposite has a rough and irregular surface. The layers are not well aligned, and some flaws are visible on the surface. It is noteworthy that the surface of the material contains a greater number of voids compared to the previous sample. This suggests that the printability of this blend is lower, as the Fe_3_O_4_ reduces the rigidity and stability of the printed structure. This composite's layer adhesion and structural integrity are also compromised, as there are some voids and cracks on the surface.

As shown in the final set of Figure 7 (g, h, and i), the nanocomposite with the highest Fe_3_O_4_ among the three nanocomposites is the PLA‐PBAT‐Fe_3_O_4_‐20%. The larger voids contribute to the overall irregularity and non‐uniformity of the surface morphology, giving it a distinct and visually striking quality. The printability of this blend is very low, as the Fe_3_O_4_ dominates the printed structure and causes severe warping and layer separation. This blend's layer adhesion and structural integrity are also very poor, as there are large gaps and cracks on the surface.

In consequence, the SEM images show that the Fe_3_O_4_ content significantly affects PLA‐PBAT's printability and printing quality, which is the primary matrix. As the Fe_3_O_4_ content increases, the printability and printing quality of PLA‐PBAT decrease. This implies a trade‐off between the printing performance and the functional properties of the PLA‐PBAT blends, which should be considered for specific application requirements. Diederichs et al.^[^ ^53^ ^]^ recommended that enhancing cohesion could be achieved by modifying the melt temperature. Increasing the melt or nozzle temperature resulted in smaller void sizes between layers. In addition to the material, the FDM print mechanism itself is also one of the main sources of microholes between rasters.^[^ ^54^, ^55^ ^]^

### 3.3 Morphology

**Figure 8:**
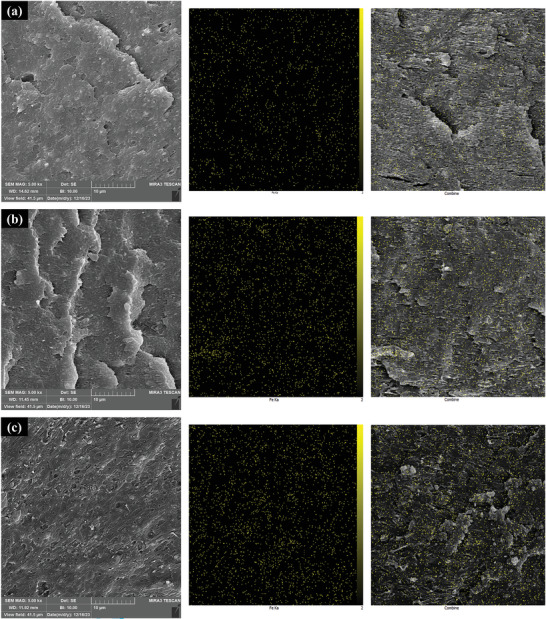
SEM morphology analysis of PLA‐PBAT‐Fe_3_O_4_ nanocomposites with different weight percentages of magnetic nanoparticles: a) 10%, b) 15%, and c) 20%.

The morphology development in immiscible polymer blends is influenced by the interplay of process variables (such as temperature, deformation types, and rate), as well as the properties of blend components (including composition, viscosity ratio, interfacial tension, continuous phase viscosity, and elasticity of the components).^[^ ^56^ ^]^ As depicted in **Figure** **8**, the morphology images of the tensile fractured surface of the PLA‐PBAT‐Fe_3_O_4_ nanocomposites are depicted at a magnification of ×5000. The PLA‐PBAT blend displays a characteristic “sea‐island” morphology, with PBAT spheres distributed within the PLA matrix. The observed phase separation of the PLA and PBAT blends suggests a lack of strong interfacial adhesion due to their immiscibility, a phenomenon documented in other studies.^[^ ^13^, ^57^, ^58^, ^59^ ^]^ The addition of Fe_3_O_4_ nanocomposite from 10 to 20 wt% does not disrupt this inherent phase separation, although the discrete domains experience selective structural modifications.

Evident from the micrographs, the Fe_3_O_4_ nanoparticles, highlighted as yellow dots, are distributed throughout the material without significant accumulation at any particular point. This indicates favorable interactions between the nanofillers and PBAT over PLA. The uniform distribution of Fe_3_O_4_ nanoparticles throughout the material, ensures consistent reinforcement across the PBAT domains. This even distribution minimizes the risk of stress concentration points, enhancing the overall mechanical performance. As a result, the PBAT phases adopt a roughened morphology, with the uniformly embedded Fe_3_O_4_ nanoparticles serving as effective reinforcement sites. This leads to improved load distribution within the PBAT regions. Meanwhile, the continuous PLA phase remains largely unaffected, maintaining its morphology due to the limited interaction with the nanoparticles.

With rising Fe_3_O_4_ content from 10 to 20 wt%, the density of nanoparticles housed within the PBAT appears to increase progressively. This suggests that the extent of phase localization and mechanical property enhancements are likely dependent on the nanoparticle loading. The changes in domain morphology also imply that the interfacial adhesion between discrete and continuous phases may be altered. Compositional variance would need to be correlated with quantitative measurements of mechanical reinforcement to ascertain these structure‐performance interrelationships.

Accordingly, the addition of Fe_3_O_4_ nanocomposite induces selective morphological modifications and reinforcement of the discrete PBAT domains in PLA/PBAT blends. The nanoparticle localization patterns indicate preferential filler‐matrix interactions that could enable targeted enhancements of the phase‐separated morphology. Enhancing compatibilization can decrease the interfacial tension that stabilizes the dispersed phase, resulting in the formation of smaller sizes and ultimately refining the dispersed phase.^[^ ^59^ ^]^

### 3.4 Mechanical Properties

**Figure 9:**
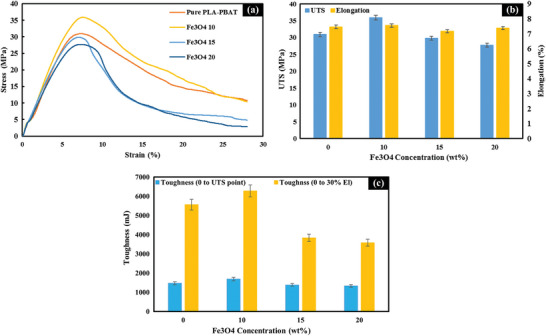
a) Stress‐Strain curves for various Fe_3_O_4_ concentrations, b) Fe_3_O_4_ concentration (wt%) correlation with UTS and uniform elongation, c) Correlation of Fe_3_O_4_ concentration with toughness: Analysis of toughness ranges from 0 to the UTS point and 0 to 30% elongation.

The stress‐strain curve in **Figure** **9**a depicts the mechanical performance of PLA‐PBAT nanocomposites as Fe_3_O_4_ nanoparticle loading increases. This property is crucial for addressing the inherent brittleness of PLA. In addition, **Table** **A1** displays quantitative data concerning the mechanical properties of all samples. As evidence, introducing magnetic nanoparticles leads to both beneficial enhancements and detrimental reductions dependent on the integrated concentration. 3D printing constructs objects through the sequential deposition of material layers. The interlayer bonding in 3D‐printed objects may exhibit lower strength when contrasted with the cohesive structure of conventionally manufactured components. This diminished adhesion between layers has the potential to lead to a decrease in the overall tensile strength of the printed objects.^[^ ^60^ ^]^

The addition of Fe_3_O_4_ leads to noticeable improvements in UTS and elongation. The nanocomposite with 10 wt% Fe_3_O_4_ shows optimal performance, with a UTS of 35.89 MPa and uniform elongation of 7.55%, representing increases of 16% and 1.1%, respectively, compared to the unfilled PLA‐PBAT blend. This enhancement results from strong interfacial interactions that restrict chain mobility and dissipate stress. In comparison, previous studies reported UTS values between 28.2 and 32.3 MPa and uniform elongation of 7.42% for PLA/PBAT (70/30 wt%) without Fe_3_O_4_,^[^ ^22^, ^61^, ^62^ ^]^ highlighting the significant reinforcement provided by the nanoparticles and the method of 3D printing in this study.

Further additions of Fe_3_O_4_ beyond 10 wt% lead to incremental declines in mechanical properties. The 15 and 20 wt% nanocomposites display reduced UTS values of 29.8 and 27.7 MPa, along with uniform elongations of 7.16% and 7.37%, respectively. These values fall below those of the pure PLA‐PBAT blend, indicating a detrimental effect at higher nanoparticle concentrations. Two factors contribute to this trend. First, nanoparticle agglomerations become increasingly likely, acting as flaws that facilitate premature failure. Second, the viscosity of the melt mixture rises with higher filler content, introducing difficulties in dispersion and defects from improper mixing.

It is important to note that the bar charts in Figure 9b confirm the optimal 10 wt% Fe_3_O_4_ loading for simultaneous enhancement of UTS and uniform elongation percentage. Toughness values, which are represented in Figure 9c, follow comparable trends. Toughness represents the ability of a material to absorb mechanical energy without fracturing.^[^ ^22^ ^]^ The nanocomposite with 10 wt% Fe_3_O_4_ loading achieves the highest toughness values of 1691.10 mJ (measured to the UTS point) and 6281.70 mJ (measured to 30% elongation). These values represent increases of 15% and 13%, respectively, compared to the unfilled PLA‐PBAT blend. In contrast, higher Fe_3_O_4_ concentrations lead to reductions in toughness. The 15 and 20 wt% nanocomposites exhibit decreased toughness values of 1385.97 and 1335.16 mJ (to UTS) and 3833.89 and 3582.05 mJ (to 30% elongation), respectively. These values are notably lower than those of the pure PLA‐PBAT blend, indicating a significant loss of energy absorption capacity at higher nanoparticle loadings.

As a result, the mechanical property trends observed in PLA‐PBAT‐Fe_3_O_4_ nanocomposites reveal a critical concentration threshold at 10 wt% loading. This optimal concentration yields a 16% increase in UTS and 15% increase in toughness compared to the pure blend, likely due to effective stress transfer between nanoparticles and the polymer matrix. However, higher loadings (15 and 20 wt%) result in property degradation, with values falling below those of the pure blend. This decline may be attributed to nanoparticle agglomeration and increased melt viscosity, compromising material integrity. These findings underscore the importance of precise concentration control in nanocomposite fabrication.

### 3.5 Shape Memory Effect (SME)

**Figure 10:**
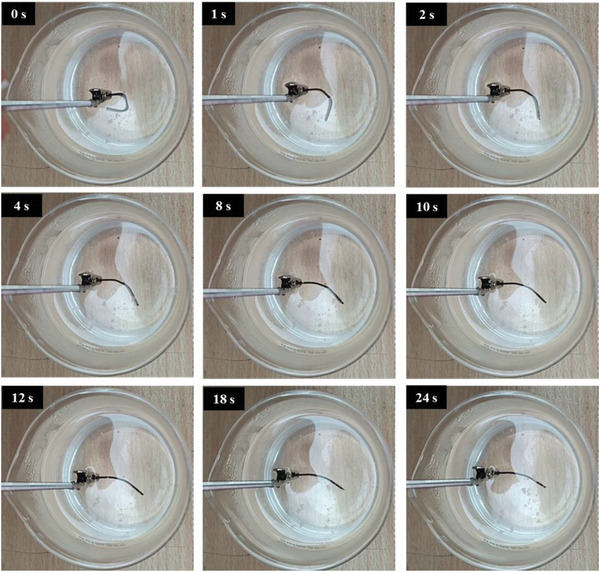
Shape recovery analysis in a hot water bath for Fe_3_O_4_‐10%.

**Figure 11:**
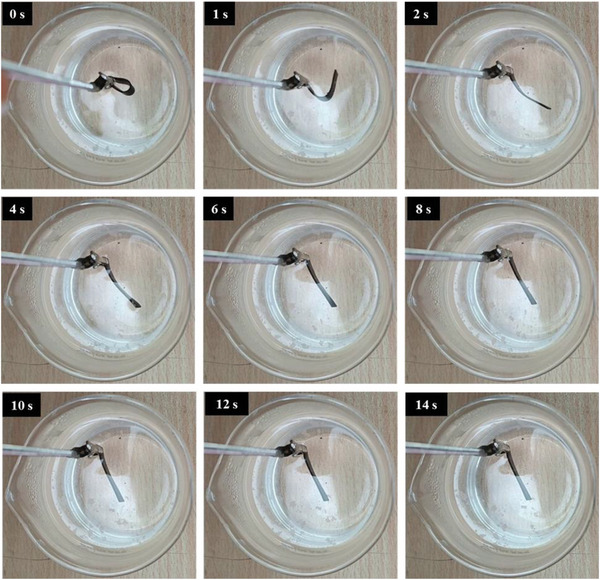
Shape recovery analysis in a hot water bath for Fe_3_O_4_‐15%.

**Figure 12:**
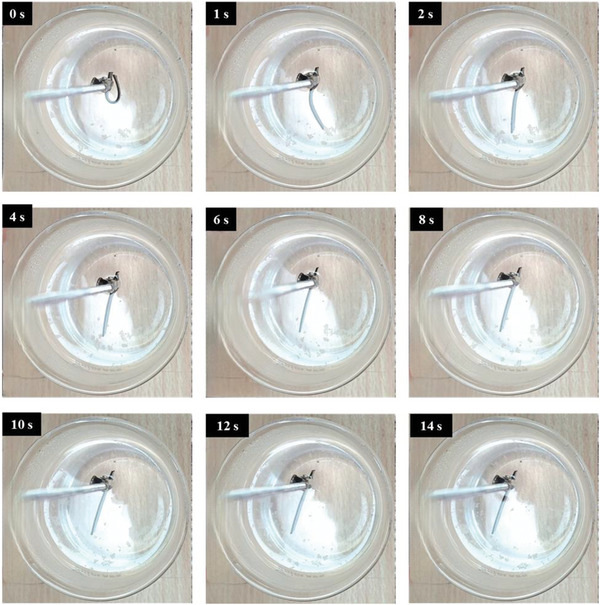
Shape recovery analysis in a hot water bath for Fe_3_O_4_‐20%.

**Figure 13:**
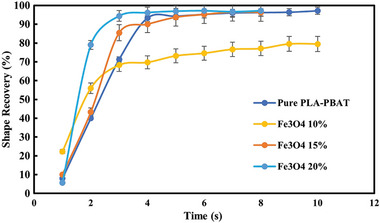
Temporal evolution of shape recovery (%) in a hot water bath: A comparative graph with time.

The recovery process of all three composites and their thermal response are presented in **Figures** 10, 11, 12. The temporary shape of the specimens is visible in the 0 s of all Figures. According to these figures, almost 100% shape recovery is observed in all samples, but the nanocomposites with different Fe_3_O_4_ contents have different recovery times. For example, Fe_3_O_4_‐10% takes more than 18 s to recover its original shape. In contrast, Fe_3_O_4_‐15% and Fe_3_O_4_‐20% samples show significantly faster recovery times, achieving more than 90% of their original shape in 4 and 3 s, respectively. This underscores the notable characteristic of the Fe_3_O_4_‐15% and Fe_3_O_4_‐20% samples: their rapid shape recovery and quick response to heat. **Figure** **13** and **Table** **A2** show the comparison of the shape recovery ratio among the specimens, which were obtained from Eq. 1. It is important to highlight that the thermal conversion efficiency increases with the content of Fe_3_O_4_, but this trend is observed not from pure PLA‐PBAT to 10% Fe_3_O_4_, but rather from 10% to 20%. Hence, the Fe_3_O_4_‐10% sample exhibits the lowest, while the Fe_3_O_4_‐20% sample has the highest shape recovery rate in the hot water bath. These findings demonstrate that the higher the weight percentage of Fe_3_O_4_, the more rapid the heat‐response of the fabricated PLA‐PBAT‐Fe_3_O_4_ nanocomposites.

**Figure 14:**
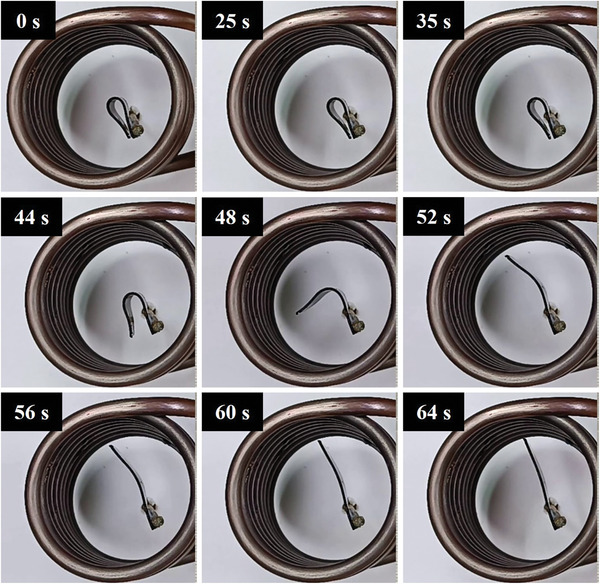
Shape recovery analysis in a magnetic field for Fe_3_O_4_‐10%.

**Figure 15:**
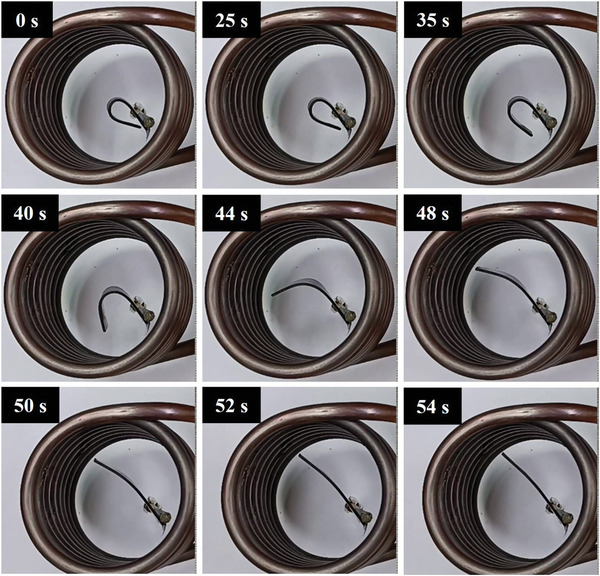
Shape recovery analysis in a magnetic field for Fe_3_O_4_‐15%.

**Figure 16:**
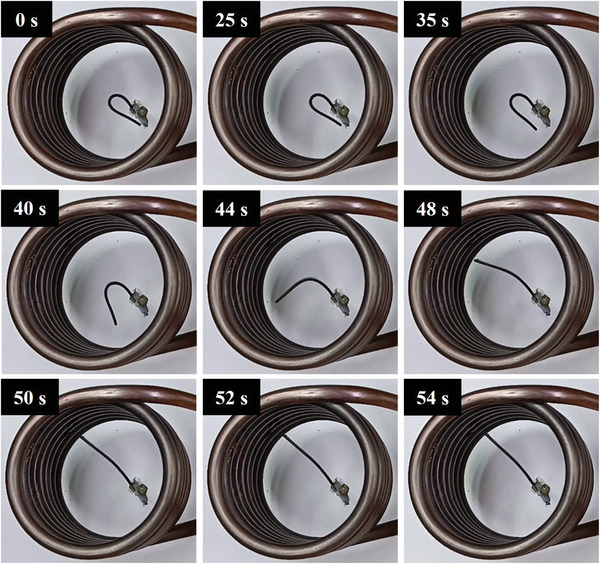
Shape recovery analysis in a magnetic field for Fe_3_O_4_‐20%.

**Figure 17:**
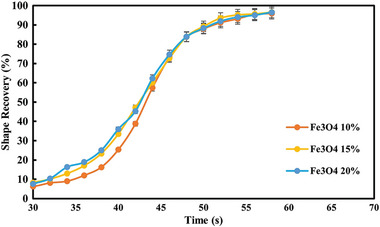
Temporal evolution of shape recovery (%) in a magnetic field: A comparative graph with time.

In the second test, shape recovery under alternating magnetic field stimulation was tested for each nanocomposite to evaluate the response of the printed PLA‐PBAT‐Fe_3_O_4_ nanocomposites to the magnetic field over time in terms of their shape memory behavior. The images depicting shape recovery over time are presented in **Figures** 14, 15, 16. SMPs containing magnetic particles within their polymer matrix can be activated by a magnetic field remotely, enabling contactless and safe actuation.^[^ ^63^ ^]^ Magnetic nanoparticles oscillate within an alternating magnetic field, producing thermal energy that facilitates alterations in shape.^[^ ^41^ ^]^ The polymer heated up internally when exposed to an alternating magnetic field, initiating the magneto‐inductive recovery process. The shape recovery process, along with the thermal distribution and the instantaneous shapes, is illustrated in Figure 4. The location and the magnetic field were consistent for all PLA‐PBAT‐Fe_3_O_4_ composites. The specimens, which were bent into a temporary shape, slowly regained their original rectangular shape under the magnetic field. For instance, the sample with 10% Fe_3_O_4_ starts to quickly recover in 44 s, while the samples with 15% and 20% Fe_3_O_4_ do so in ≈40 s. The samples with 10%, 15%, and 20% Fe_3_O_4,_ respectively, take ≈64, 54, and 56 s to fully recover. They exhibit a similar pattern as the previous test in the hot water bath, except they take longer to fully recover their original shape. In **Figure** **17** and **Table** **A3**, the amount of shape recovery at different times is quantitatively presented. As can be seen, all three nanocomposites show almost similar shape recovery behavior and magnetic response, with the difference that the recovery start time completely depends on the weight percentage of magnetic nanoparticles. It is worth mentioning that the samples with higher Fe_3_O_4_ content recover their original shape faster due to their magnetic characteristic. The blend matrix can quickly distribute the heat from the magnetic field due to the high thermal conductivity of Fe_3_O_4_.^[^ ^64^, ^65^ ^]^

The above analysis shows that PLA has been widely utilized as an SMP with shape recovery ability. A small amount of PBAT is added as a toughening agent, which shows a ductile behavior at room temperature to preserve PLA from permanent damage during the deformation and shape recovery process. A noteworthy finding is that the amorphous region of PLA in the glassy state became mobile and formed a temporary shape when the temperature exceeded the T_g_ of PLA. The Fe_3_O_4_ particles dispersed in the specimen are activated by the magnetic field, and they produce and transfer a large amount of heat and energy to the polymer matrix. The specimens regain their original shape in a specific time span when the temperature in the samples reaches the T_g_ of PLA again, as the elastic energy stored in the glassy molecular chain is released. Thus, the process by which PLA‐PBAT‐Fe_3_O_4_ composites undergo magneto‐thermal conversion has been explained and inferred.

## 4 Conclusion

This work pioneers the development and advanced characterization of 3D/4D printable PLA‐PBAT nanocomposites reinforced with varying weight percentages (10%, 15%, and 20%) of Fe_3_O_4_ nanoparticles. The multifunctional performance enhancements spanning thermomechanical properties, tensile attributes, printability, and rapid thermal and magnetic actuation have not been reported previously. The key conclusions arising from this study are summarized as follows:

- The DMTA and SEM results confirm that the PLA‐PBAT blends are two‐phase and immiscible, with DMTA further revealing that the transition temperatures of PBAT and PLA are crucial for determining material compatibility. The intensity and magnitude of peaks in the graphs indicated the volume percentage of each material in the mixture. Achieving closer transition temperatures enhanced compatibility, while the glassy state and rubbery plateau regions signify different mechanical properties at varying temperature ranges.
- The incorporation of 10 wt% Fe_3_O_4_ nanoparticles into PLA/PBAT blends significantly improved mechanical properties, including a 16% increase in UTS (35.89 MPa) and a 15% increase in toughness (1691.10 mJ) compared to the unfilled blend. This improvement stems from strong nanoparticle‐polymer interactions, enhancing stress transfer and energy dissipation. However, further increases in Fe_3_O_4_ concentration (15 and 20 wt%) led to reduced mechanical performance, with UTS dropping to 29.8 and 27.7 MPa, respectively, due to nanoparticle agglomeration and elevated melt viscosity. This highlights the importance of maintaining an optimal nanoparticle loading for enhanced material performance.
- SEM images indicated that all 3D‐printed blends exhibit high printing quality, likely due to the enhanced control of material flow provided by the new pneumatic system on the FDM printer. However, as the Fe_3_O_4_ content increased from 10% to 20%, SEM micrographs revealed increasing roughness of PBAT domains, suggesting enhanced interfacial interactions and targeted property augmentations. Despite the high printing quality, the printability, printing quality, and layer adhesion of PLA‐PBAT decreased significantly with higher Fe_3_O_4_ content. This decline is attributed to increased surface voids and enhanced flexibility, ultimately diminishing stability.
- Increasing the Fe_3_O_4_ concentration accelerated both the thermally and magnetically activated shape memory response and recovery kinetics of the PLA‐PBAT‐Fe_3_O_4_ nanocomposites. All samples exhibit almost 100% shape recovery, but the time it takes for recovery varies depending on the Fe_3_O_4_ content in the nanocomposites. Higher Fe_3_O_4_ concentrations reduced recovery times due to improved thermal conductivity and more efficient heat distribution within the polymer matrix. This enabled tunable 4D printability and actuation performance based on tailored nanoparticle additions, especially in thermal and magnetic environments.

Overall, the magnetite nanofillers imparted noticeable yet balanced enhancements of PLA‐PBAT blend attributes, significantly increasing both mechanical and shape memory properties without compromising printability. The work highlights the promise of this multifunctional reinforcement approach in developing high‐performance, sustainable materials and 4D‐printed structures with remote activation and actuation capabilities.

## Conflict of Interest

The authors declare no conflict of interest.

## Data Availability

The data that support the findings of this study are available from the corresponding author upon reasonable request.

## 1

**Table A1:** Quantitative outcomes derived from the tensile test.

Samples	Tensile Test			
	UTS [MPa]	Uniform Elongation [%]	Toughness [mJ]	
			0 to UTS point	0 to 30% El
Pure PLA‐PBAT	30.93	7.47	1470.07	5565.80
Fe_3_O_4_‐10%	35.89	7.55	1691.10	6281.70
Fe_3_O_4_‐15%	29.81	7.16	1385.97	3833.89
Fe_3_O_4_‐20%	27.72	7.37	1335.16	3582.05

**Table A2:** Shape recovery (%) results derived from the shape memory test in a hot water bath.

Sample	Time [s]					
	1	2	3	4	6	8
Pure PLA‐PBAT	7.97	40.00	71.29	93.33	95.23	96.22
Fe_3_O_4_‐10%	22.31	55.98	68.30	69.73	74.59	77.10
Fe_3_O_4_‐15%	10.07	43.30	85.46	90.01	95.22	96.32
Fe_3_O_4_‐20%	5.70	79.03	94.42	96.20	97.18	97.22

**Table A3:** Shape recovery (%) results derived from the shape memory test in a magnetic field.

Sample	Time [s]					
	30	34	40	46	52	58
Fe_3_O_4_‐10%	6.27	9.03	25.36	73.32	91.16	96.58
Fe_3_O_4_‐15%	8.33	13.00	33.49	72.73	93.54	96.63
Fe_3_O_4_‐20%	7.65	16.36	36.00	74.68	91.94	96.58

## References

[1] A. Karimi , D. Rahmatabadi , M. Baghani , Polym. 2024, 16, 831. 10.3390/polym16020267 PMC10820913 38257066

[2] M. Bodaghi , A. Sadooghi , M. Bakhshi , S. J. Hashemi , K. Rahmani , M. K. Motamedi , Adv. Mater. Interfaces 2023, 10, 2300337.

[3] M. Grosjean , L. Guth , S. Déjean , C. Paniagua , B. Nottelet , Adv. Mater. Interfaces 2023, 10, 2300066.

[4] A. K. K. Padinjareveetil , M. Pumera , Adv. Mater. Interfaces 2023, 10, 2201734.

[5] B. Wittbrodt , J. M. Pearce , Addit. Manuf. 2015, 8, 110.

[6] L. Wang , W. M. Gramlich , D. J. Gardner , Polym. 2017, 114, 242.

[7] V. E. Kuznetsov , A. N. Solonin , O. D. Urzhumtsev , R. Schilling , A. G. Tavitov , Polym. 2018, 10, 313. 10.3390/polym10030313 PMC6414919 30966348

[8] D. Garlotta , J. Polym. Environ. 2001, 9, 63.

[9] X. Zhao , H. Hu , X. Wang , X. Yu , W. Zhou , S. Peng , RSC Adv. 2020, 10, 13316. 35492128 10.1039/d0ra01801e PMC9051451

[10] D. Rahmatabadi , K. Soltanmohammadi , M. Aberoumand , E. Soleyman , I. Ghasemi , M. Baniassadi , K. Abrinia , M. Bodaghi , M. Baghani , Phys. Scr. 2024, 99, 025013. 10.3390/polym14245446 PMC9787995 36559813

[11] Z. Yang , H. Peng , W. Wang , T. Liu , J. Appl. Polym. Sci. 2010, 116, 2658.

[12] R. Al‐Itry , K. Lamnawar , A. Maazouz , Polym. Degrad. Stab. 2012, 97, 1898.

[13] R. Al‐Itry , K. Lamnawar , A. Maazouz , Rheol. Acta. 2014, 53, 501.

[14] J. Andrzejewski , J. Cheng , A. Anstey , A. K. Mohanty , M. Misra , ACS Sustainable Chem. Eng. 2020, 8, 6576.

[15] M. Bianchi , A. Dorigato , M. Morreale , A. Pegoretti , Polym. 2023, 15, 881. 10.3390/polym15040881 PMC9963890 36850164

[16] T. Zhang , W. Han , C. Zhang , Y. Weng , Polym. Degrad. Stab. 2021, 183, 109455.

[17] A. Karimi , D. Rahmatabadi , M. Baghani , Polym. 2024, 16, 267. 10.3390/polym16020267 PMC10820913 38257066

[18] B. Wang , Y. Jin , K. Kang , N. Yang , Y. Weng , Z. Huang , S. Men , E‐Polymers 2020, 20, 39.

[19] H. He , G. Wang , M. Chen , C. Xiong , Y. Li , Y. Tong , Materials 2020, 13, 2094. 32369995 10.3390/ma13092094 PMC7254402

[20] Y. Fu , G. Wu , X. Bian , J. Zeng , Y. Weng , Molecules 2020, 25. 32872416 10.3390/molecules25173946 PMC7504808

[21] D. Rahmatabadi , M. Khajepour , A. Bayati , K. Mirasadi , M. Amin Yousefi , A. Shegeft , I. Ghasemi , M. Baniassadi , K. Abrinia , M. Bodaghi , M. Baghani , Eur. Polym. J. 2024, 216, 113289.

[22] J. Mathew , J. P. Das , M. Tp , S. Kumar , J. Polym. Res. 2022, 29, 474.

[23] E. Sritham , P. Phunsombat , J. Chaishome , MATEC Web Conf 2018, 192, 03014.

[24] P. H. M. Cardoso , R. R. T. P. Coutinho , F. R. Drummond , M. do N. da Conceição , R. M. . da S. M Thiré , Macromol. Symp. 2020, 394, 2000157.

[25] W. Chen , C. Qi , Y. Li , H. Tao , Radiat. Phys. Chem. 2021, 180, 109239.

[26] S. K. Leist , D. Gao , R. Chiou , J. Zhou , Virtual Phys. Prototyp. 2017, 12, 290.

[27] C. M. González‐Henríquez , M. A. Sarabia‐Vallejos , J. Rodriguez‐Hernandez , Prog. Polym. Sci. 2019, 94, 57.

[28] F. Momeni , S. M. M. Hassani N , X. Liu , J. Ni , Mater. Des. 2017, 122, 42.

[29] J. Bai , G. Bu , J. Adv. Manuf. Sci. Technol. 2022, 2, 202200.

[30] H. Meng , G. Li , Polym. 2013, 54, 2199.

[31] M. Y. Razzaq , M. Behl , A. Lendlein , MRS Adv. 2019, 4, 1057.

[32] M. Behl , M. Y. Razzaq , A. Lendlein , Adv. Mater. 2010, 22, 3388. 20574951 10.1002/adma.200904447

[33] S. Ahadian , A. Khademhosseini , Regen. Biomater. 2018, 5, 125. 29977595 10.1093/rb/rby007 PMC6007551

[34] S. Miao , W. Zhu , N. J. Castro , J. Leng , L. G. Zhang , Tissue Eng. – Part C Methods 2016, 22, 952. 28195832 10.1089/ten.tec.2015.0542 PMC5079413

[35] W. Zhao , F. Zhang , J. Leng , Y. Liu , Compos. Sci. Technol. 2019, 184, 107866.

[36] H. Pandey , S. S. Mohol , R. Kandi , Mater. Lett. 2022, 329, 133238.

[37] A. Melocchi , N. Inverardi , M. Uboldi , F. Baldi , A. Maroni , S. Pandini , F. Briatico‐Vangosa , L. Zema , A. Gazzaniga , Int. J. Pharm. 2019, 559, 299. 30707934 10.1016/j.ijpharm.2019.01.045

[38] S. Li , Y. Huan , B. Zhu , H. Chen , M. Tang , Y. Yan , C. Wang , Z. Ouyang , X. Li , J. Xue , W. Wang , J. Mater. Sci. Mater. Med. 2022, 33, 2. 10.1007/s10856-021-06609-4 PMC8702412 34940930

[39] B. I. Oladapo , S. O. Ismail , O. M. Ikumapayi , P. G. Karagiannidis , Colloids Surf., B. 2022, 216, 112583. 10.1016/j.colsurfb.2022.112583 35662072

[40] M. De Wild , S. Dany , C. John , F. Schuler , Curr. Dir. Biomed. Eng. 2020, 6, 3.

[41] F. Zhang , L. Wang , Z. Zheng , Y. Liu , J. Leng , Compos. Part A Appl. Sci. Manuf. 2019, 125, 105571.

[42] H. Liu , F. Wang , W. Wu , X. Dong , L. Sang , Compos. Part B Eng. 2023, 248, 110382.

[43] A. Bayati , D. Rahmatabadi , I. Ghasemi , M. Khodaei , M. Baniassadi , K. Abrinia , M. Baghani , Mater. Lett. 2024, 361, 136075.

[44] T. Liu , K. Chen , J. Vinyl Addit. Technol. 2024, 30, 983.

[45] Y. Liao , C. Liu , B. Coppola , G. Barra , L. Di Maio , L. Incarnato , K. Lafdi , Polym. 2019, 11, 1487. 10.3390/polym11091487 PMC6781044 31547357

[46] A. Rodríguez‐Panes , J. Claver , A. Camacho , Materials 2018, 11, 1333. 30071663 10.3390/ma11081333 PMC6119930

[47] J. Kiendl , C. Gao , Compos. Part B Eng. 2020, 180, 107562.

[48] A. Adedeji , A. M. Jamieson , Polym. 1993, 34, 5038.

[49] J. D. Badia , L. Santonja‐Blasco , A. Martínez‐Felipe , A. Ribes‐Greus , Charact. Polym. Blends Miscibility, Morphol. Interfaces 2015, 9783527331, 365.

[50] B. Tan , L. S. Stephens , Tribol. Int. 2019, 140, 105870.

[51] N. Sabyrov , A. Abilgaziyev , M. H. Ali , Int. J. Adv. Manuf. Technol. 2020, 108, 603.

[52] S. Garzon‐Hernandez , A. Arias , D. Garcia‐Gonzalez , Compos. Part B Eng. 2020, 201, 108373.

[53] E. V. Diederichs , M. C. Picard , B. P. Chang , M. Misra , D. F. Mielewski , A. K. Mohanty , ACS Omega 2019, 4, 20297. 31815232 10.1021/acsomega.9b02795 PMC6893943

[54] Y. Tao , F. Kong , Z. Li , J. Zhang , X. Zhao , Q. Yin , D. Xing , P. Li , A Review on Voids of 3D Printed Parts by Fused Filament Fabrication, Elsevier, Netherlands 2021.

[55] D. Rahmatabadi , M. Aberoumand , K. Soltanmohammadi , E. Soleyman , I. Ghasemi , M. Baniassadi , K. Abrinia , M. Bodaghi , M. Baghani , Macromol. Mater. Eng. 2023, 2300114. 10.3390/polym14245446 PMC9787995 36559813

[56] L. C. Arruda , M. Magaton , R. E. S. Bretas , M. M. Ueki , Polym. Test. 2015, 43, 27.

[57] S. Su , M. Duhme , R. Kopitzky , Materials 2020, 13, 4897. 33142823 10.3390/ma13214897 PMC7662590

[58] J. Yeh , C. Tsou , C. Huang , K. Chen , C. Wu , W. Chai , J. Appl. Polym. Sci. 2010, 116, 680.

[59] Y. Lyu , Y. Chen , Z. Lin , J. Zhang , X. Shi , Compos. Sci. Technol. 2020, 200, 108399.

[60] C. G. Amza , A. Zapciu , G. Constantin , F. Baciu , M. I. Vasile , Polym. 2021, 13, 562. 10.3390/polym13040562 PMC7918060 33668615

[61] M. Bianchi , A. Dorigato , M. Morreale , A. Pegoretti , Polym. 2023, 15, 881. 10.3390/polym15040881 PMC9963890 36850164

[62] D. Rahmatabadi , M. Khajepour , A. Bayati , K. Mirasadi , Eur. Polym. J. 2024, 216, 113289.

[63] Q. Ze , X. Kuang , S. Wu , J. Wong , S. M. Montgomery , R. Zhang , J. M. Kovitz , F. Yang , H. J. Qi , R. Zhao , Adv. Mater. 2020, 32, 1906657. 10.1002/adma.201906657 31814185

[64] Z. Wu , J. Chen , Q. Li , D. H. Xia , Y. Deng , Y. Zhang , Z. Qin , Materials 2021, 14, 2013. 33923696 10.3390/ma14082013 PMC8074025

[65] D. Rahmatabadi , K. Mirasadi , A. Bayati , M. Khajepour , M. Baniassadi , K. Abrinia , M. Baghani , I. Ghasemi , M. Bodaghi , Appl. Mater. Today 2024, 40, 102361.

